# Time-Dynamic analysis of sex-specific NREM sleep disturbance induced by social isolation among adolescent mice

**DOI:** 10.1038/s41398-026-03895-w

**Published:** 2026-02-13

**Authors:** Shuangyan Li, Xuxuan Ma, Yu Jiang, Haicheng Guo, Panyue Zhong, Leqin Fang, Jihong Liu, Bin Zhang

**Affiliations:** 1 Department of Psychiatry, Sleep Medicine Center, Nanfang Hospital, Southern Medical University, Guangzhou, China; 2 Guangdong - Hong Kong - Macao Greater Bay Area Center for Brain Science and Brain - Inspired Intelligence, Southern Medical University, Guangzhou, China; 3 Institute of Brain Disease, Nanfang Hospital of Southern Medical University, Guangzhou, China; 4 Key Laboratory of Mental Health of the Ministry of Education, Southern Medical University, Guangzhou, China

**Keywords:** Psychiatric disorders, Molecular neuroscience

## Abstract

Sleep disturbances are more prevalent in women than in men during adulthood. However, since age-related changes in sleep and the consequences of sleep disturbances can occur as early as adolescence, it remains poorly understood whether these disturbances exhibit a similar sex-specific pattern during adolescence, and what the underlying molecular mechanisms may be. Male and female mice were subjected to social isolation stress starting at postnatal day 21 (P21), and electroencephalography (EEG) was monitored during isolation period. We then employed whole-brain transcriptomic analysis and Mfuzz enrichment analysis to identify temporal and sex-specific molecular responses, dynamic gene expression patterns, and key pathways during isolation period. Male mice exhibited decreased non-rapid eye movement (NREM) sleep duration after 2, 3, and 4 weeks of isolation, while female mice did not show these disturbances after 2 and 3 weeks, but did after 4 weeks of isolation. This suggested a sex-specific pattern of sleep disturbances during adolescence, which differs from those observed in adulthood. Moreover, the decreased NREM sleep in isolated male mice was related to sensory, metabolic, and immune systems after 2, 3, and 4 weeks of isolation, respectively. While the reduction in NREM duration in female mice after 4 weeks of isolation was associated with their energy metabolism and amino acid metabolism. We found a sex-specific pattern of sleep disturbances during adolescence, with male mice being more susceptible to social isolation stress, which may be linked to early sensory system responses in isolated male mice and later-stage amino acid metabolism and energy imbalance in isolated female mice. Our findings provide insights into gender-specific interventions for sleep disorders during adolescence and underscore the importance of considering both temporal and sex differences in stress-related sleep research.

## Introduction

Sleep architecture, characterized by the cyclical alternation between non-rapid eye movement (NREM) and rapid eye movement (REM) sleep, serves as a critical biomarker of physiological health and neurodevelopment [1, 2]. Sleep architecture exhibits age-specific patterns across the lifespan, with distinct changes occurring during key developmental stages [3]. Sleep disorders are highly prevalent among adults, with research consistently showing that women are more likely to experience sleep disturbances than men during adulthood. It suggested a gender-based disparity in the prevalence of sleep disorders [4]. However, while age-related changes in sleep and the consequences of sleep disturbances can begin as early as adolescence, it remains unclear whether similar sex-specific patterns of sleep disturbances emerge during adolescence, and the underlying molecular mechanisms are still poorly understood.

Adolescence is a sensitive period for stress-induced mental disorders, as well as a critical period for the development of stable sleep patterns and structure. Sleep architecture during adolescence not only reflects the brain’s sensitivity to stress [5, 6], but also predicts the onset and progression of mental disorders [7, 8] and physical diseases [9, 10]. Lewin et al. found that rats subjected to early life trauma showed a reduction in spindle activity during early life, and fragmented NREM sleep in later years [11]. However, most of these studies did not report gender differences, which limit our understanding of the potential mechanisms of the relationship between stress-induced sleep patterns and gender differences among adolescent mammals. Meanwhile, previous studies have suggested that the duration of stress during adolescence is closely linked to the severity and type of mental disorders, with longer exposure to stress leading to more complex or severe symptoms [12]. This further emphasizes the need for long-term longitudinal tracking of sleep structure changes and gender differences throughout adolescence in animal models.

In our study, we employed a social isolation (SI) model among adolescent mice to evaluate a time-dynamic analysis of alterations in their sleep patterns, and incorporated omics data to investigate the temporal impact of sex differences on sleep disorders during SI. Additionally, we employed whole-brain transcriptomic analysis and Mfuzz enrichment analysis to identify sex-specific molecular mechanisms.

## Materials and methods

### Mice

Three-week-old male and female C57BL/6 J mice (purchased from the Guangdong Medical Laboratory Animal Center) were used for the study. The mice were housed in standard laboratory cages under a 12 h light/dark cycle (lights on at 8:00 AM) in a temperature-controlled room (21-25 °C). Food and water were provided ad libitum. Littermate mice were randomly assigned to experimental groups. In the transcriptomic sequencing experiment, at least 3 animals were included in each group, and in the behavioral tests, each group included at least 7 subjects. All experiments were conducted in accordance with the Regulations for the Administration of Affairs Concerning Experimental Animals (China) and approved by the Southern Medical University Animal Ethics Committee. All experimental protocols were conducted following institutional guidelines.

### Animal model

**Fig. 1:**
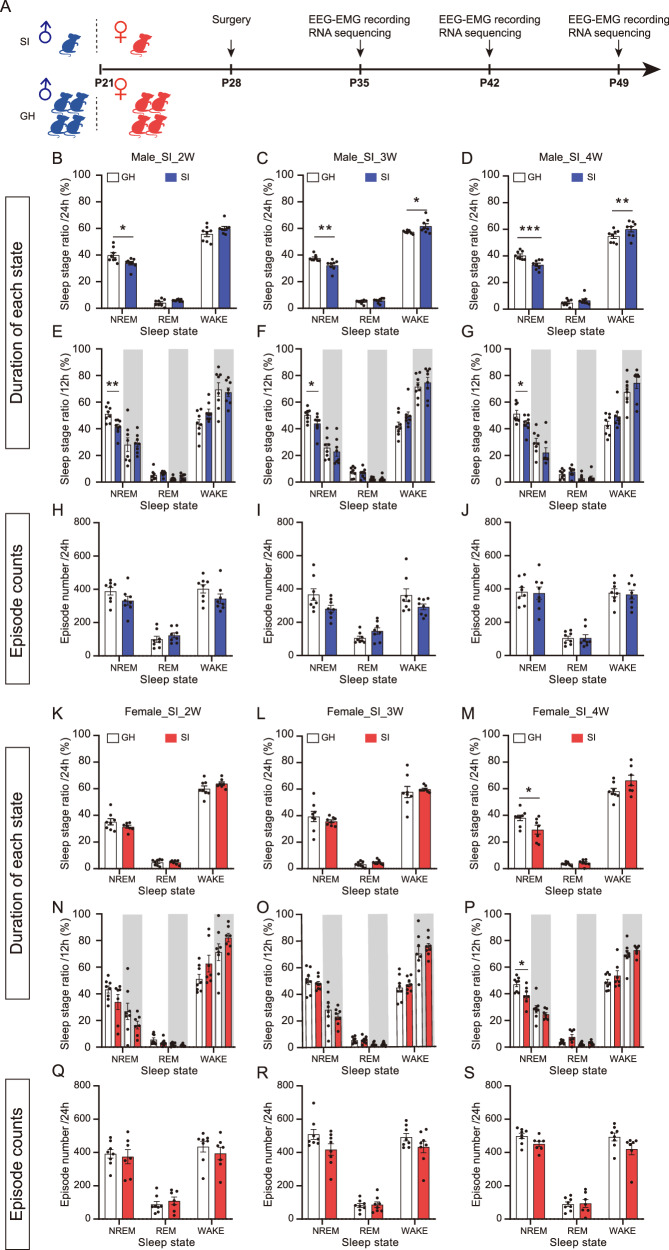
SI reduces the duration of NREM sleep in male mice starting from week 2, while female mice exhibit similar effects at week 4. **A** Timeline of EEG-EMG recording and transcriptome sequencing during SI in adolescence. **B-D** Percentage of total recording time spent in NREM, REM, and Wake states over 24 h in male mice after 2 weeks (**B**), 3 weeks (**C**), and 4 weeks (**D**) of SI. **E-G** Percentage of total recording time spent in NREM, REM, and Wake states over 12 h light/dark phases in male mice after 2 weeks (**E**), 3 weeks (**F**), and 4 weeks (**G**) of SI. White indicates the light phase and gray indicates the dark phase. **H–J** Episode numbers of each sleep state in male mice over 24 h after 2 weeks (**H**), 3 weeks (**I**), and 4 weeks (**J**) of SI. **K-M** Percentage of total recording time spent in NREM, REM, and Wake states over 24 h in female mice after 2 weeks (**K**), 3 weeks (**L**), and 4 weeks (**M**) of SI. **N-P** Percentage of total recording time spent in NREM, REM, and Wake states over 12 h light/dark phases in female mice after 2 weeks (**N**), 3 weeks (**O**), and 4 weeks (**P**) of SI. White indicates the light phase and gray indicates the dark phase. **Q–S** Episode numbers of each sleep state in female mice over 24 h after 2 weeks (**Q**), 3 weeks (**R**), and 4 weeks (**S**) of SI. Data are presented as mean ± SEM. Each dot represents one mouse. For most groups, n = 8 per group; female SI group at 2 weeks and 4 weeks: n = 7. Statistical significance was assessed using an unpaired two-tailed Student’s t-test. *p < 0.05, **p < 0.01, ***p < 0.001.

The experimental paradigm is illustrated in Fig. 1. The study included an experimental group of C57BL/6 J mice, which were housed individually in single cages starting at postnatal day 21 for 2, 3, and 4 weeks to establish a social isolation model (SI), and a control group of mice housed in group cages (GH), with 4 mice per cage. Each week, both groups underwent 24 h EEG-EMG monitoring to assess sleep-wake patterns. After monitoring, whole-brain tissue samples were collected for RNA extraction, and transcriptome sequencing was performed. The data were then analyzed using the Mfuzz package to identify gene expression patterns over time and investigate how SI affects gene regulation in the brain.

### EEG-EMG electrodes implantation

The EEG-EMG electrodes consisted of four stainless steel screws and two Teflon-coated silver leads. Surgery was performed to implant the electrodes. Mice were deeply anesthetized with an intraperitoneal injection of pentobarbital sodium (75 mg/kg). The mouse head was shaved, and the skull surface was exposed through a midline scalp incision. The periosteal tissue over the skull was removed. Four small holes (1 mm in diameter) were drilled above the frontal and parietal regions. The EEG electrodes were implanted into these holes and fixed with dental cement. Two electrodes for EMG recording were placed on the nuchal muscle and stabilized. After the dental cement had fully set and dried, the animals were returned to their cages to recover for one week before recording. Mice that exhibited a postoperative body weight loss greater than 20% or developed severe complications were excluded from subsequent analyses.

### EEG-EMG analysis

The EEG and EMG signals were amplified, filtered with a high-pass filter above 0.5 Hz, and digitized at a 1000 Hz resolution using a tethered data acquisition system (Medusa, Bio-Signal Technologies, China). The sleep stages in the recordings were scored by AI-driven software, Lunion Stage, developed by LunionData (China). The EEG-EMG data were analyzed in 4 s epochs and categorized into three stages: non-rapid eye movement (NREM) sleep, rapid eye movement (REM) sleep, and wakefulness (Wake). The scored results were reviewed, and manual adjustments were made as necessary. NREM sleep was characterized by low-frequency δ waves (0.5-4 Hz) with high amplitude in EEG signals, accompanied by low-amplitude EMG activity. REM sleep was characterized by high-frequency θ waves (4-8 Hz) with low amplitude in EEG signals and a flat EMG pattern. During wakefulness, the EEG displayed high-frequency (8-50 Hz), low-amplitude activity, while the EMG displayed high-amplitude muscle activity. Based on the scored sleep stages, statistical analysis was performed on the different vigilance states in the various experimental groups.

### Transcriptome sequencing

After the 24 h EEG-EMG monitoring, mice were euthanized in the waking state, and whole-brain tissue was rapidly collected from both groups. The collected tissues were immediately frozen in liquid nitrogen and stored at −80 °C until RNA extraction. Total RNA was isolated, and high-throughput RNA sequencing was performed to assess gene expression differences between the experimental and control groups at each time point (2, 3, and 4 weeks).

### RNA sequencing analysis

Raw sequencing reads were mapped to the reference genome using HISAT2, and gene-level expression was quantified as raw counts using HTSeq. To ensure comparability of gene expression profiles across samples, raw counts were normalized to transcripts per million (TPM). Normalized expression data were log2-transformed and used for downstream analysis. Differential expression analysis was performed using the DESeq2 package (version 1.18.1). The resulting P-values were adjusted using Benjamini and Hochberg’s method to control the false discovery rate. Differentially expressed genes (DEGs) were defined as those with an adjusted P-value < 0.05 and |log_2_(FoldChange)| > 1. The GOSeq package (version 1.34.1) was used to identify Gene Ontology (GO) terms that annotate the list of enriched genes (adjusted P-value < 0.05). Kyoto Encyclopedia of Genes and Genomes (KEGG) analysis was used to analyze the enriched pathways of the candidate genes (adjusted P-value < 0.05). The top 30 GO terms for biological process (BP), cellular component (CC), and molecular function (MF), as well as the top 20 KEGG signaling pathways, were selected for further investigation.

### Mfuzz analysis

Differential gene expression between female and male mice under SI for different weeks was analyzed using the Mfuzz package (version 2.66.0) in R (version 4.4.2). The fuzzy c-means algorithm was applied to cluster genes with similar expression patterns over time. This analysis grouped the differentially expressed genes into distinct clusters based on their expression profiles during the isolation periods. The optimal number of clusters was determined using the elbow method. This clustering approach enabled the identification of gene groups that exhibited similar regulatory patterns, offering insights into how SI influenced gene expression in both female and male mice at various time points [13].

To further interpret the biological significance of clusters identified from the Mfuzz analysis, we focused on the gene expression patterns within each cluster. These clusters represent dynamic changes in gene expression during the SI periods, with each cluster reflecting a distinct temporal pattern of gene regulation. The optimal number of clusters was determined using the elbow method, ensuring the most meaningful partitioning of the gene expression data. Rather than treating these clusters as categorical labels, they were considered as dynamic gene-expression modules that exhibit time-dependent fluctuations. By selecting clusters that showed significant deviations from baseline at various time points (SI2W, SI3W, SI4W), we identified the gene sets most responsive to SI, which were further analyzed to link the molecular changes to the observed alterations in sleep patterns.

### Quantitative real-time PCR

Brain tissue samples were dissected and prepared after selection, with the samples stored directly in Trizol reagent (Invitrogen, 15596026). RNA extraction was performed according to the manufacturer’s instructions. cDNA was synthesized using the PrimeScript RT Reagent Kit (Accurate Biotechnology, AG11705). Quantitative real-time PCR (qPCR) was performed with SYBR Green Master Mix (Accurate Biology, AG11718) on a QuantStudio 6 Flex Real-Time PCR System (Applied Biosystems). Primers are listed in Supplementary Table 2. Expression of target genes was normalized to 18S rRNA as an endogenous control.

### Statistical analysis

Behavioral data were recorded by observers blinded to experimental conditions, and subsequent data analysis was performed by investigators blinded to group identity. Data normality was assessed using the Shapiro-Wilk test, and homogeneity of variances was evaluated using Levene’s test before each statistical test. In the experimental data, two-tailed unpaired Student’s t-test was used to compare the means of two independent samples using SPSS 22 software (SPSS, Chicago, IL). The mean values shown in the text and figures are expressed as the mean ± standard error of the mean (SEM). *p* < 0.05 was considered statistically significant, and GraphPad Prism 10 (La Jolla, CA) was used to draw the graphs.

## Results

### Sex-specific NREM sleep alterations during SI across adolescence

To investigate the effects of SI on the sleep patterns of male and female mice, we housed the mice either in groups or singly starting from P21. Subsequently, we conducted electroencephalography (EEG) monitoring at 1, 2, 3, and 4 weeks of isolation (Fig. 1A). EEG monitoring showed that, while 1-week SI did not affect sleep structure (Supplementary fig. S1), male mice subjected to 2, 3, and 4 weeks of SI exhibited a significant reduction in NREM sleep duration compared to the GH group (Fig. 1B-D). Specifically, during the light phase, the proportion of NREM sleep in male mice decreased significantly after 2, 3, and 4 weeks of isolation (Fig. 1E-G). The number of episodes of NREM, REM, and Wake states during the 24 h or 12 h phases showed no differences between the GH and SI groups in male mice after 2, 3, and 4 weeks of isolation (Fig. 1H-J; Supplementary fig. S3A-C).

In contrast, female mice did not exhibit a reduction in NREM sleep duration after 2 or 3 weeks of isolation, but showed a reduction after 4 weeks of isolation (Fig. 1K-M). During the light phase, no reduction in NREM sleep was observed after 2 or 3 weeks of isolation (Fig. 1N, O), but a significant reduction was detected after 4 weeks of isolation (Fig. 1P). Analysis of the episode numbers in each sleep stage showed no differences between the GH and SI groups in female mice after 2, 3, and 4 weeks of isolation (Fig. 1Q-S; Supplementary fig. S3D-F). The average episode duration of NREM sleep did not differ significantly both in male and female mice at any time point (Supplementary fig. S3G-H). These results suggest a sex-specific disturbance in sleep during adolescence, with male mice being more susceptible than female mice, a pattern that differs from that observed in adult mice. In addition, we also provide the total real time spent in NREM, REM, and Wake during the 24 h or 12 h phases after 1, 2, 3, and 4 weeks of isolation, respectively (Supplementary fig. S2).

### Sex-specific temporal transcriptomic responses to SI in male and female mice

**Fig. 2:**
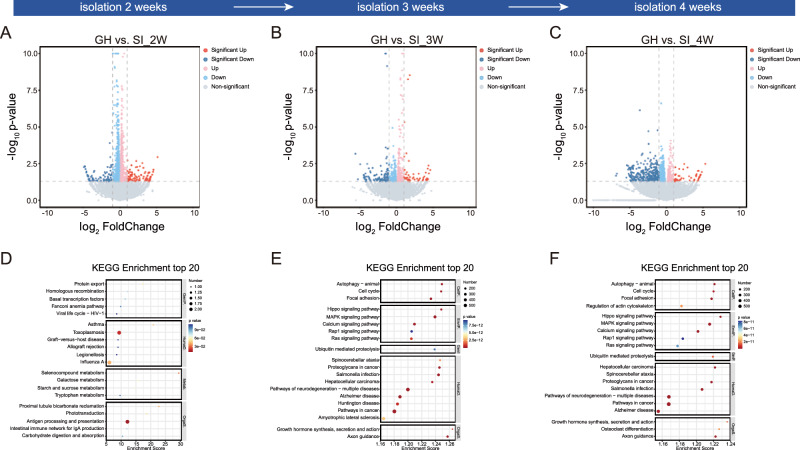
Whole-brain transcriptomic profiling of male mice under SI. **A-C** Volcano plots showing DEGs in male mice after 2, 3, and 4 weeks of isolation compared to GH controls. Red dots represent significantly upregulated genes, and blue dots represent significantly downregulated genes [cutoff: |log_2_FC | > 1, adjusted *P* < 0.05. (2 weeks: Down: Sig: 81, Up: Sig: 96; 3 weeks: Down: Sig: 102, Up: Sig: 52; 4 weeks: Down: Sig: 288, Up: Sig: 51)]. **D-F** KEGG pathway enrichment analysis of DEGs at 2, 3, and 4 weeks of isolation, showing the top 20 significantly enriched pathways. KEGG: Kyoto Encyclopedia of Genes and Genomes; GenIP.: Genetic Information Processing; HumaD.: Human Diseases; Metab.: Metabolism; OrgaS.: Organismal Systems; EnvIP.: Environmental Information Processing; CellP.: Cellular Processes.

Next, to investigate the temporal and sex-specific molecular responses to prolonged SI, we conducted a whole-brain transcriptomic analysis on both male and female mice at various time points following SI. Firstly, we performed whole-brain transcriptomic sequencing on male mice at 2, 3, and 4 weeks of isolation. As shown in the volcano plot, a total of 177 DEGs (96 upregulated and 81 downregulated) were detected in male mice subjected to 2 weeks of isolation compared to the GH controls (Fig. 2A); Similarly, 154 DEGs (52 upregulated and 102 downregulated) and 339 DEGs (51 upregulated and 288 downregulated) were detected at 3 and 4 weeks of isolation, respectively (Fig. 2B, C).

To explore the biological processes and pathways affected by SI, we performed GO and KEGG pathway analyses on DEGs from male mice at 2, 3, and 4 weeks of isolation. At 2 weeks of isolation, GO analysis revealed significant enrichment in BP related to the “RNA catabolic process” (Supplementary fig. S4A). The most affected KEGG pathway was “antigen processing and presentation” (Fig. 2D). After 3 weeks, GO terms associated with “protein transport” and “cell cycle” were enriched (Supplementary fig. S4B), while KEGG analysis identified pathways such as “MAPK signaling pathway” and “pathways of neurodegeneration-multiple diseases” (Fig. 2E). At 4 weeks, the most enriched BP terms were related to “protein transport” and “cell cycle” (Supplementary fig. S4C), and KEGG analysis revealed significant enrichment in pathways such as the “MAPK signaling pathway”, “cancer signaling pathways” and “neurodegeneration signaling pathways” (Fig. 2F).

**Fig. 3:**
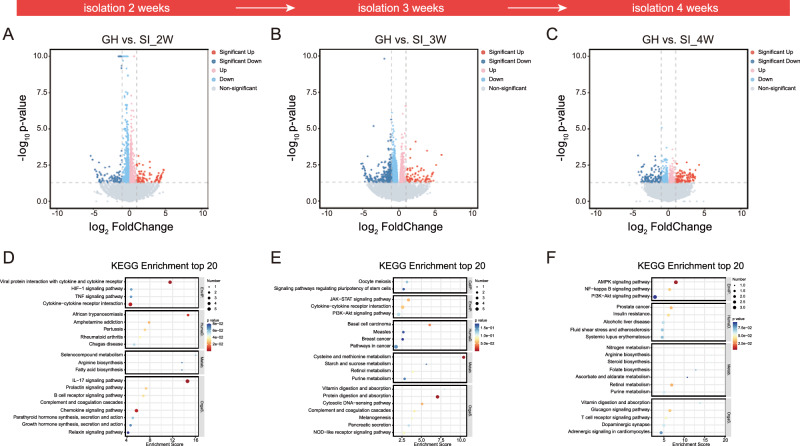
Whole-brain transcriptomic profiling of female mice under SI. **A-C** Volcano plots showing DEGs in female mice after 2, 3, and 4 weeks of isolation compared to GH controls. Red dots represent significantly upregulated genes, and blue dots represent significantly downregulated genes [cutoff: |log_2_FC | > 1, adjusted *P* < 0.05. (2 weeks: Down: Sig: 103, Up: Sig: 108; 3 weeks: Down: Sig: 271, Up: Sig: 80; 4 weeks: Down: Sig: 117, Up: Sig: 120)]. **D-F** KEGG pathway enrichment analysis of DEGs at 2, 3, and 4 weeks of isolation, showing the top 20 significantly enriched pathways. KEGG: Kyoto Encyclopedia of Genes and Genomes; GenIP.: Genetic Information Processing; HumaD.: Human Diseases; Metab.: Metabolism; OrgaS.: Organismal Systems; EnvIP.: Environmental Information Processing; CellP.: Cellular Processes.

Secondly, we identified 211 significant DEGs in female mice subjected to 2 weeks of isolation (Fig. 3A), including 108 upregulated and 103 downregulated genes. Similarly, 351 DEGs (80 upregulated and 271 downregulated) and 237 DEGs (120 upregulated and 117 downregulated) were detected at 3 and 4 weeks of isolation, respectively (Fig. 3B, C). Supplementary fig. S4D-F provide information on the results of GO annotation analysis in female mice. At 2 weeks of isolation, GO analysis revealed significant enrichment in BP terms related to “positive regulation of toll-like receptor 3 signaling pathway” and “negative regulation of plasminogen activation” (Supplementary fig. S4D). By 3 weeks, the most enriched BP terms were related to the “meiotic cell cycle” and “positive regulation of proteolysis” (Supplementary fig. S4E). At 4 weeks, the most enriched BP term was “mammary gland alveolus development” (Supplementary fig. S4F). Based on KEGG pathway analysis, the most significantly enriched pathways in female mice at 2 weeks of isolation were the “IL-17 signaling pathway” and “viral protein interaction with cytokine and cytokine receptor” (Fig. 3D). At 3 weeks, the enriched pathways included “protein digestion and absorption” and “cysteine and methionine metabolism” (Fig. 3E), while at 4 weeks, the most significantly enriched pathway was the “AMPK signaling pathway” (Fig. 3F).

### Temporal dynamics of gene expression clusters in male and female mice under SI stress

**Fig. 4:**
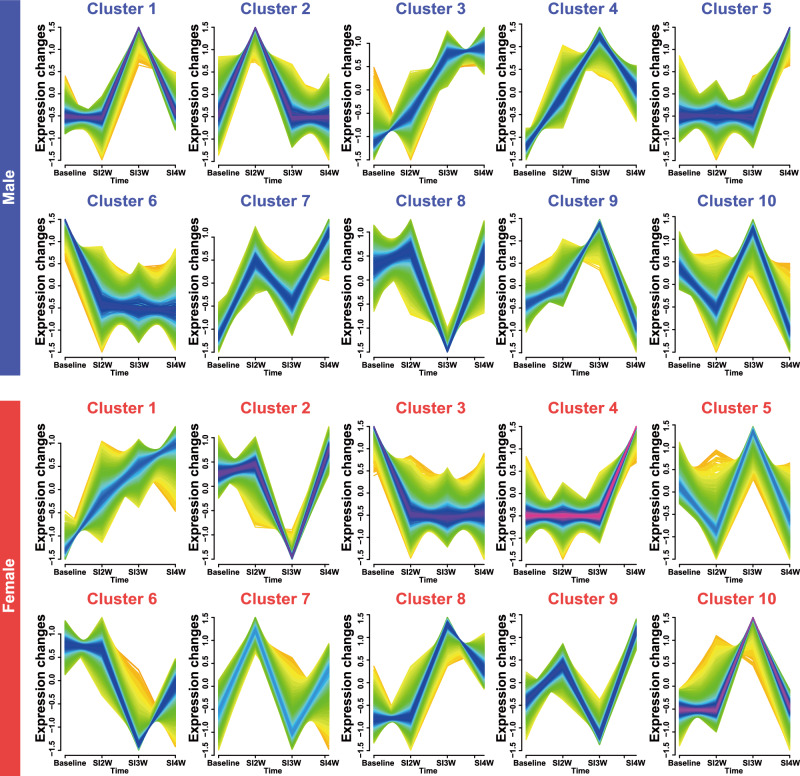
Temporal dynamics of gene expression clusters during SI identified by Mfuzz analysis. Fuzzy c-means clustering (Mfuzz) was applied separately to DEGs in male (top panel) and female (bottom panel) mice across baseline (GH2W) and SI weeks 2, 3, and 4 (SI2W, SI3W, SI4W) to identify sex-specific temporal expression patterns. Male-specific gene expression clusters: A total of 10 clusters were identified, each showing a distinct trajectory of transcriptional changes in response to prolonged SI. Female-specific gene expression clusters: A total of 10 clusters were identified, revealing dynamic shifts in gene expression over time. Each cluster represents standardized expression changes (y-axis) across different time points (x-axis), with color gradients indicating expression density. Genes within the same cluster exhibit similar temporal expression patterns, suggesting potential co-regulation.

To further explore the dynamic patterns of gene expression changes over time, we used the Mfuzz package to perform fuzzy c-means clustering on the time-course transcriptomic data. This analysis revealed distinct temporal expression profiles in both male and female mice subjected to SI, highlighting clusters of genes that were specifically upregulated or downregulated during 2-, 3-, and 4-week isolation periods. We identified 10 distinct clusters in both male and female mice, each representing a unique set of genes with specific expression patterns at particular time points. In male mice, the gene expression in Cluster 1 showed no significant differences between the 2- and 4-week isolation periods, but peaked specifically at 3-week isolation. This pattern was similarly observed in Clusters 4 and 8, suggesting that these clusters represent genes specifically responsive to 3 weeks of isolation. Gene expression in Cluster 2 peaked at the 2-week isolation mark and then declined to baseline levels at the 3- and 4-week time points. In contrast, genes in Cluster 5 showed peak expression at the 4-week isolation period (Fig. 4).

Figure 4 also presents the Mfuzz results for female mice. Cluster 7 represents genes specifically expressed after 2 weeks of isolation. Gene expression in Cluster 2 reached its lowest point at 3 weeks of isolation, while genes in Cluster 10 reached their peak expression at the same time point. These two clusters specifically reflect distinct transcriptional responses in female mice at the 3-week isolation, highlighting their unique roles in the molecular changes associated with this critical period. Genes within Cluster 4 maintained consistent expression levels at both 2- and 3-week isolation time points, but demonstrated significant upregulation specifically at the 4-week time point, indicating that these genes are uniquely associated with transcriptional changes in female mice at 4 weeks of isolation.

### Identification and functional enrichment of key genes associated with NREM sleep disturbance in male mice during SI

**Fig. 5:**
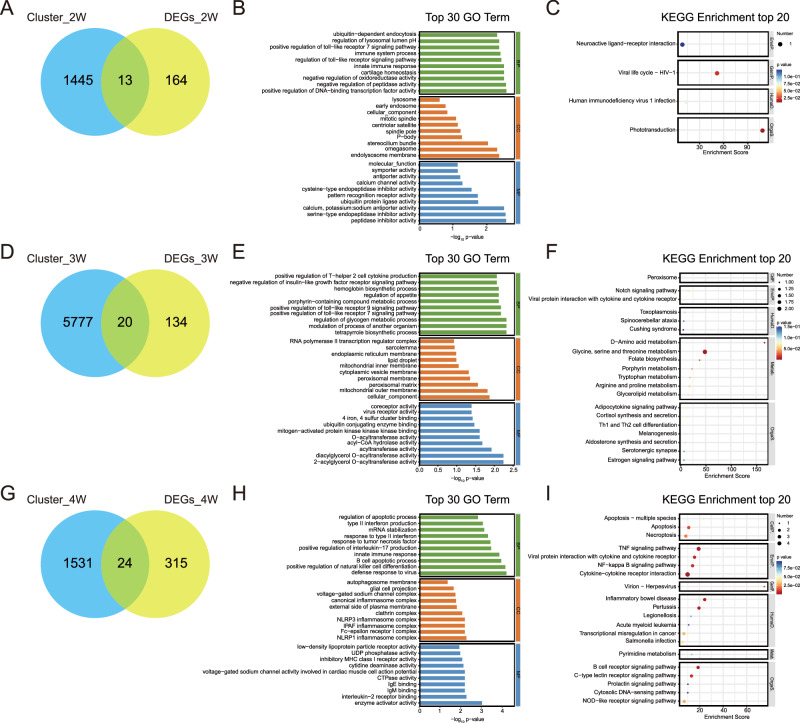
Functional enrichment analysis of DEGs identified within Mfuzz clusters in male mice. **A, D, G** Venn diagrams showing the intersection between genes identified in Mfuzz clustering analysis and DEGs at week 2 (**A**), 3 (**D**), and 4 (**G**) of SI in male mice. The overlapping regions represent genes shared between clusters and DEGs, which were further analyzed for functional enrichment. **B, E, H** GO enrichment analysis of intersecting genes at week 2 (**B**), 3 (**E**), and 4 (**H**), highlighting the top 30 enriched biological terms across BP, CC, and MF. **C, F, I** KEGG pathway enrichment analysis of intersecting genes at week 2 (**C**), 3 (**F**), and 4 (**I**), displaying the top 20 significantly enriched pathways.

To further identify key genes and their associated signaling pathways at each time point during SI, we performed an overlap analysis between the DEGs sets and the gene sets corresponding to each time point, as identified by Mfuzz. We visualized the overlapping genes using Venn diagrams. At 2-week SI time point, we identified 13 key genes involved in the emergence of NREM sleep disturbance in male mice, as shown in Fig. 5A. Additionally, GO and KEGG analyses revealed that these 13 key genes were primarily enriched in BP terms associated with the “positive regulation of DNA-binding transcription factor activity” (Fig. 5B). The most significantly enriched pathway was “phototransduction” (Fig. 5C), which showed a strong preference for organismal systems, particularly the sensory system (Supplementary fig. S5A).

After 3 weeks of isolation, 20 key genes were identified as responsive to NREM sleep disturbance in male mice (Fig. 5D). These genes were mainly enriched in the following BP terms (Fig. 5E): “tetrapyrrole biosynthetic process”, “modulation of process of another organism cell body”, and “regulation of glycogen metabolic process”, among others. These processes were linked to metabolic functions such as amino acid metabolism, lipid metabolism, and metabolism of cofactors and vitamins (Fig. 5F, Supplementary fig. S5B).

Finally, after 4 weeks of isolation, 24 key genes were identified in male mice, and their biological processes were primarily enriched in “defense response to virus”, “positive regulation of natural killer cell differentiation”, “B cell apoptotic process” and “innate immune response” (Fig. 5G, H). These pathways are primarily associated with immune system functions (Fig. 5I, Supplementary fig. S5C). The relationship between these key genes and sleep-wake traits was shown in Supplementary fig. S6A-C. We conducted further analysis focusing on specific brain regions, namely the basal lateral amygdala (BLA) and the lateral hypothalamus (LH), which are closely involved in sleep-wake regulation and stress responses. We selected the top five key genes with significant fold-changes and small p-values from these regions and performed mRNA measurements. Our results indicated that the expression changes of some key genes in these specific brain regions are consistent with those observed in the whole brain transcriptomic analysis in isolated male mice (Supplementary fig. S7).

### Identification and functional enrichment of key genes associated with NREM sleep disturbance in female mice during SI

**Fig. 6:**
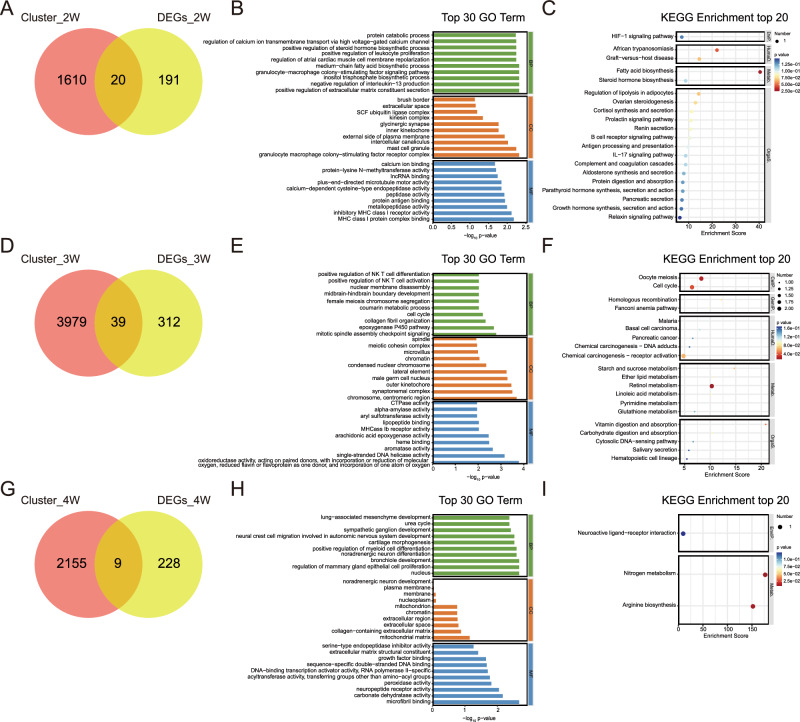
Functional enrichment analysis of DEGs identified within Mfuzz clusters in female mice. **A, D, G** Venn diagrams showing the intersection between genes identified in Mfuzz clustering analysis and DEGs at week 2 (**A**), 3 (**D**), and 4 (**G**) of SI in female mice. The overlapping regions represent genes shared between clusters and DEGs, which were further analyzed for functional enrichment. **B, E, H** GO enrichment analysis of intersecting genes at week 2 (**B**), 3 (**E**), and 4 (**H**), highlighting the top 30 enriched biological terms across BP, CC, and MF. **C, F, I** KEGG pathway enrichment analysis of intersecting genes at week 2 (**C**), 3 (**F**), and 4 (**I**), displaying the top 20 significantly enriched pathways.

To identify key genes and their associated pathways in female mice at different time points of SI, we applied the same analytical approach used for male mice. Specifically, we performed an overlap analysis between DEGs sets and the time-specific gene clusters derived from Mfuzz, with the results visualized using Venn diagrams. At 2 weeks of isolation, 20 key genes were identified in female mice, despite the absence of NREM sleep disturbance at this time point (Fig. 6A). Interestingly, GO and KEGG analyses revealed that these genes were significantly enriched in the “inositol trisphosphate biosynthetic process” and the “fatty acid biosynthesis” pathways, suggesting a potential role in lipid metabolism. The remaining KEGG pathways were primarily associated with the endocrine and immune systems (Fig. 6B, C; Supplementary fig. S5D).

After 3 weeks of isolation, 39 key genes were identified in female mice, primarily enriched in the “retinol metabolism” and “vitamin digestion and absorption” pathways (Fig. 6D-F). These pathways were associated with metabolic functions and the digestive system within organismal system, respectively (Supplementary fig. S5E).

After 4 weeks of isolation, 9 key genes were identified as linked to NREM sleep disturbance in female mice (Fig. 6G). GO analysis revealed significant enrichment in biological process related to “noradrenergic neuron differentiation” (Fig. 6H). Meanwhile, the results of KEGG indicated that the primary enriched pathways were “nitrogen metabolism”, and “arginine biosynthesis”, which were primarily involved in energy metabolism and amino acid metabolism functions (Fig. 6I). These findings suggest a strong involvement of metabolism responses in female mice after prolonged SI (Supplementary fig. S5F). Overall, these results indicate that metabolic processes play a significant role in mediating sleep disturbances induced by prolonged SI in female mice. The relationship between these key genes and sleep-wake traits was shown in Supplementary fig. S6D-F. We also conducted further analysis focusing on specific brain regions, namely the BLA and the LH, which are closely involved in sleep-wake regulation and stress responses. We selected the top five key genes with significant fold-changes and small p-values from these regions and performed mRNA measurements. Our results indicated that the expression changes of some key genes in these specific brain regions are consistent with those observed in the whole brain transcriptomic analysis, with few genes, such as *Cblif* in female mice, exhibited aggregate effect (Supplementary fig. S8).

## Discussion

Previous studies have highlighted sex-specific differences in responses to SI across various physiological systems, including hippocampal plasticity [14], cardiovascular reactivity [15], affective vulnerability [16, 17], accelerated aging [18], dendritic spine remodeling [19], and neural circuit reorganization [20]. Our study further extended SI to the sleep architecture based on this sex-specific animal model. Firstly, we found that adolescent male mice exhibited a reduction in NREM sleep duration after 2 weeks of isolation. Secondly, the NREM sleep disturbance observed in adolescent male mice after 2 weeks of isolation was associated with early activation of sensory systems. In adolescent female mice, the decrease in NREM sleep duration after 4 weeks of isolation was associated with metabolic disruptions. Although both sexes experienced a reduction in NREM sleep, the underlying regulatory mechanisms differed.

Adolescence represents a critical developmental window with heightened vulnerability to psychiatric disorders [21, 22]. SI during this period exacerbates mental health risks, with the duration of isolation contributing to distinct behavioral phenotypes. Specifically, in adolescent male mice, 2 weeks of isolation led to anxiety-like behaviors [23], 3 weeks of isolation resulted in depressive-like symptoms [16], and 4 weeks of isolation strengthened the retention of fear memories [24] and autism-like behaviors [25]. All of these researches highlight the importance of the length of isolation in shaping behavioral outcome. Interestingly, our research revealed that regardless of the duration of SI, adolescent male mice similarly exhibited a reduction in NREM sleep duration, but not in REM phase. In contrast, adolescent female mice only began to show a reduction in NREM sleep after 4 weeks of isolation. This may be due to the sensitivity of NREM sleep to chronic stress, which ultimately leads to sleep reduction [7, 26]. Moreover, NREM sleep is associated with the regulation of hypothalamic-pituitary-adrenal (HPA) axis [27], neurotransmitters [28, 29], and the immune system [30], all of which are influenced by SI [17, 31]. In contrast, the regulation of REM sleep remains relatively independent, potentially serving as a protective factor under stress conditions [32]. These findings suggest that NREM sleep may serve as a biomarker of stress responses. Additionally, the delayed decrease in NREM sleep after 4 weeks of isolation in adolescent female mice, compared to male mice, underscores the different sensitivities to SI. Sex also plays a crucial role in the relationship between NREM sleep and SI, with previous studies emphasizing gender differences in stress responses [33]. Estrogen in female mice may provide a potential protective role in stress responses [34, 35].What causes adolescent male mice to develop NREM sleep disturbance earlier than female mice, and to maintain consistent NREM sleep disturbance under dynamic changes during different periods of SI? To address this, we identified key genes with regulatory roles that vary over time during SI using Mfuzz time-series analysis. Pathway enrichment analysis showed activation of sensory systems (phototransduction pathways) at week 2 of SI, metabolic signaling pathways at week 3 of SI, and immune-related pathways and metabolic regulation at week 4 of SI.The sensory systems are responsible for receiving physical stimuli. Previous studies have shown that recovery of NREM sleep in male mice is worse than in female mice after physical stimulation [36, 37]. Additionally, behavioral manifestations in male mice, including locomotor activity, anxiety levels, and pentobarbital-induced sleep, were more sensitive to the effects of SI [38]. Another study indicated that in the fear conditioning test, after 3 weeks of isolation, male mice became more susceptible to a prolonged freezing behavior in response to foot-shock stimulation, whereas female mice did not [16]. This suggests that male mice are more vulnerable to external physical stimuli and develop NREM sleep disturbance earlier than female mice. A human transcriptomics-based study demonstrated that long-term social loneliness induces changes closely associated with inflammatory factors [39], aligning with our findings. However, prior study did not address sex-specific differences in these responses, and research on the dynamic temporal changes underlying these sex differences remains limited. Overall, sensory systems may serve as a potential target for interventions aimed at sleep disturbances in male mice, advancing the understanding of sex-specific sleep regulation and its molecular mechanisms.

To investigate the factors contributing to the delayed onset of sleep disturbances in female mice after SI, we focused on the 4-week time point of SI, which is primarily associated with metabolism and energy balance in female mice. Our research suggests that adolescent female mice may resist the detrimental effects of SI from weeks 2 to 3 through sustained activation of immune and lipid metabolism pathways. This finding is consistent with previous studies showing that female animals exhibit greater resilience to chronic stress, which is associated with immune regulation [40]. Clinical research indicates that women have a metabolic advantage in controlling blood lipids, potentially protecting them from the detrimental effects of obstructive sleep apnea [41]. Furthermore, the lower weight gain observed in female mice under chronic stress [42–44] supports the finding that metabolic regulation plays a critical role in their stress adaptation. These initial protective mechanisms may become overwhelmed, leading to eventual disruption of NREM sleep when female mice experience long-term stress. Lipid metabolism or immune modulation may represent potential preventive strategies for female sleep disorders in the future. In our study, after 4 weeks of social isolation, both male and female mice exhibited reduced NREM sleep duration. Transcriptomic analysis revealed that the changes in males were mainly enriched in immune-related pathways, whereas in females, they were mainly associated with metabolic dysregulation. Previous studies have demonstrated that NREM sleep was closely linked to both immune [45, 46] and metabolic functions [47, 48]. Therefore, we suspect that social isolation may impair NREM sleep in males primarily through immune dysfunction, while in females, metabolic imbalance following stress may play a more critical role. We note that these associations are correlative, and further studies will be required to establish causal mechanisms.

In conclusion, our study first revealed significant sex-specific differences in the temporal dynamics of sleep architecture under SI conditions. While the molecular mechanisms underlying sex differences in the relationship between SI and sleep disturbances are not yet fully understood, whole-brain transcriptomic analysis has provided a broad perspective on the gene expression changes induced by SI. However, it does not explore the specific roles of individual brain regions, which will be addressed in future studies. The potential roles of hormones, particularly the estrous cycle in females, as well as genetic, and epigenetic factors should also be explored in further studies. This research establishes a foundation for developing targeted interventions aimed at alleviating the negative impacts of SI on adolescent mental health. Additionally, it offers valuable insights into the development of gender-specific mouse models for studying sleep disorders.

## Supplementary information

Supplementary information

## Data Availability

Data and code will be made available on request.
