# Supplementary material for: Time-Dynamic analysis of sex-specific NREM sleep disturbance induced by social isolation among adolescent mice
Source: Transl Psychiatry. 2026 Feb 13;16:165. doi: 10.1038/s41398-026-03895-w (PMC13021962; doi:10.1038/s41398-026-03895-w)
Supplement: Supplementary file 1 — Supplementary information [file 41398_2026_3895_MOESM1_ESM.docx]

**Supplementary Information**

**Time-Dynamic Analysis of Sex-Specific NREM Sleep Disturbance Induced by Social Isolation Among Adolescent Mice**

Shuangyan Li et al.


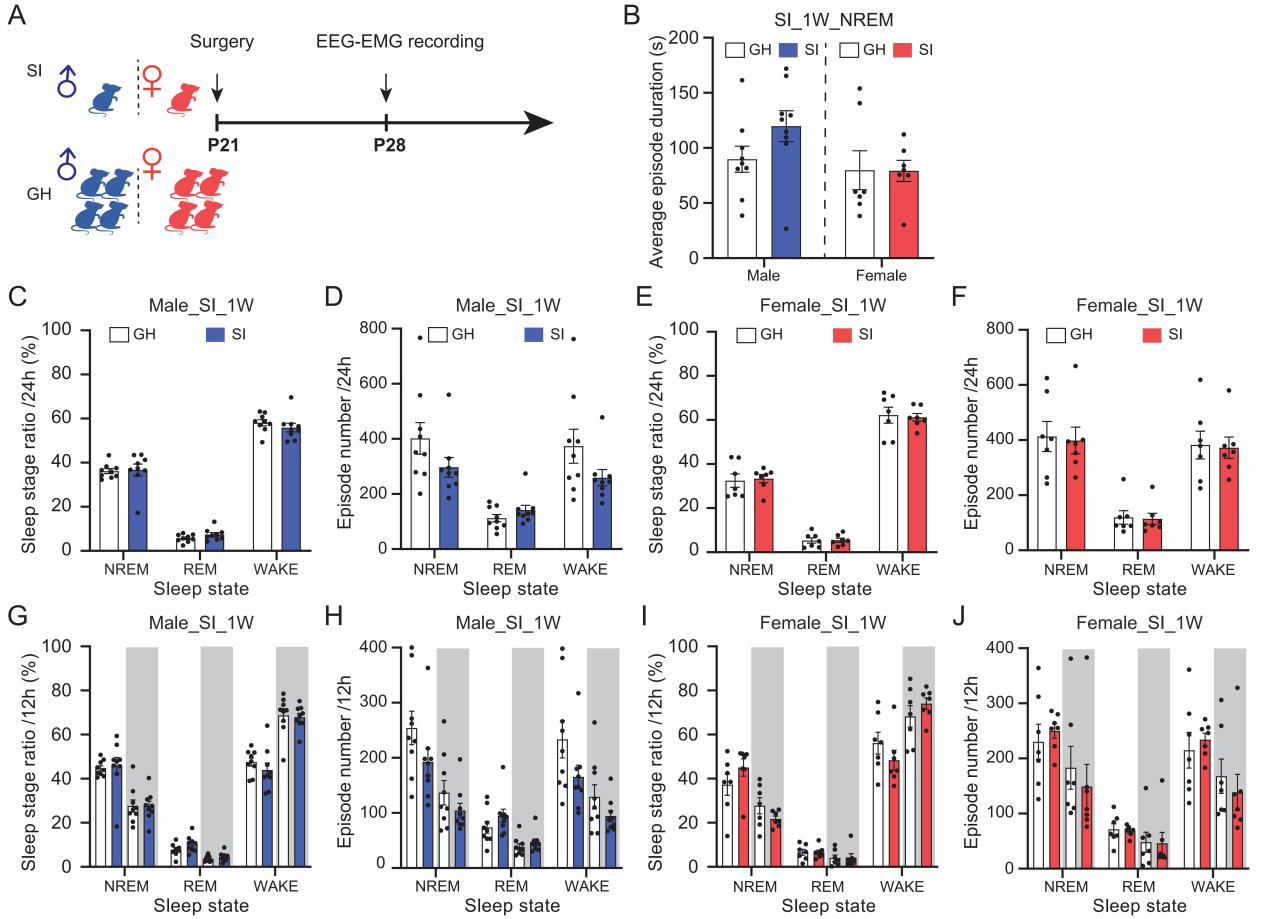


**Supplementary Fig.1. Social isolation for 1 week does not alter sleep structure in either male or female mice.**

(A) Timeline of EEG - EMG recording after 1 week of social isolation in both male and female mice.

(B) Average episode duration of NREM sleep in male and female mice after 1 week of SI.

(C) Percentage of total recording time spent in NREM, REM, and Wake states over 24 hours in male mice.

(D) Episode counts of each sleep state in male mice over 24 hours.

(E) Percentage of total recording time spent in NREM, REM, and Wake states over 24 hours in female mice.

(F) Episode counts of each sleep state in female mice over 24 hours.

(G) Percentage of total recording time spent in NREM, REM, and Wake states over 12-hour light/dark phases in male mice. White indicates the light phase and gray indicates the dark phase.

(H) Episode counts of each sleep state in male mice over 12-hour light/dark phases.

(I) Percentage of total recording time spent in NREM, REM, and Wake states over 12-hour light/dark phases in female mice.

(J) Episode counts of each sleep state in female mice over 12-hour light/dark phases.

Data are presented as mean ± SEM. Each dot represents one mouse. Male groups: n = 9 per group; female groups: n = 7 per group. Statistical significance was assessed using an unpaired two-tailed Student’ s t-test. **p* < 0.05, ***p* < 0.01, ****p* < 0.001.


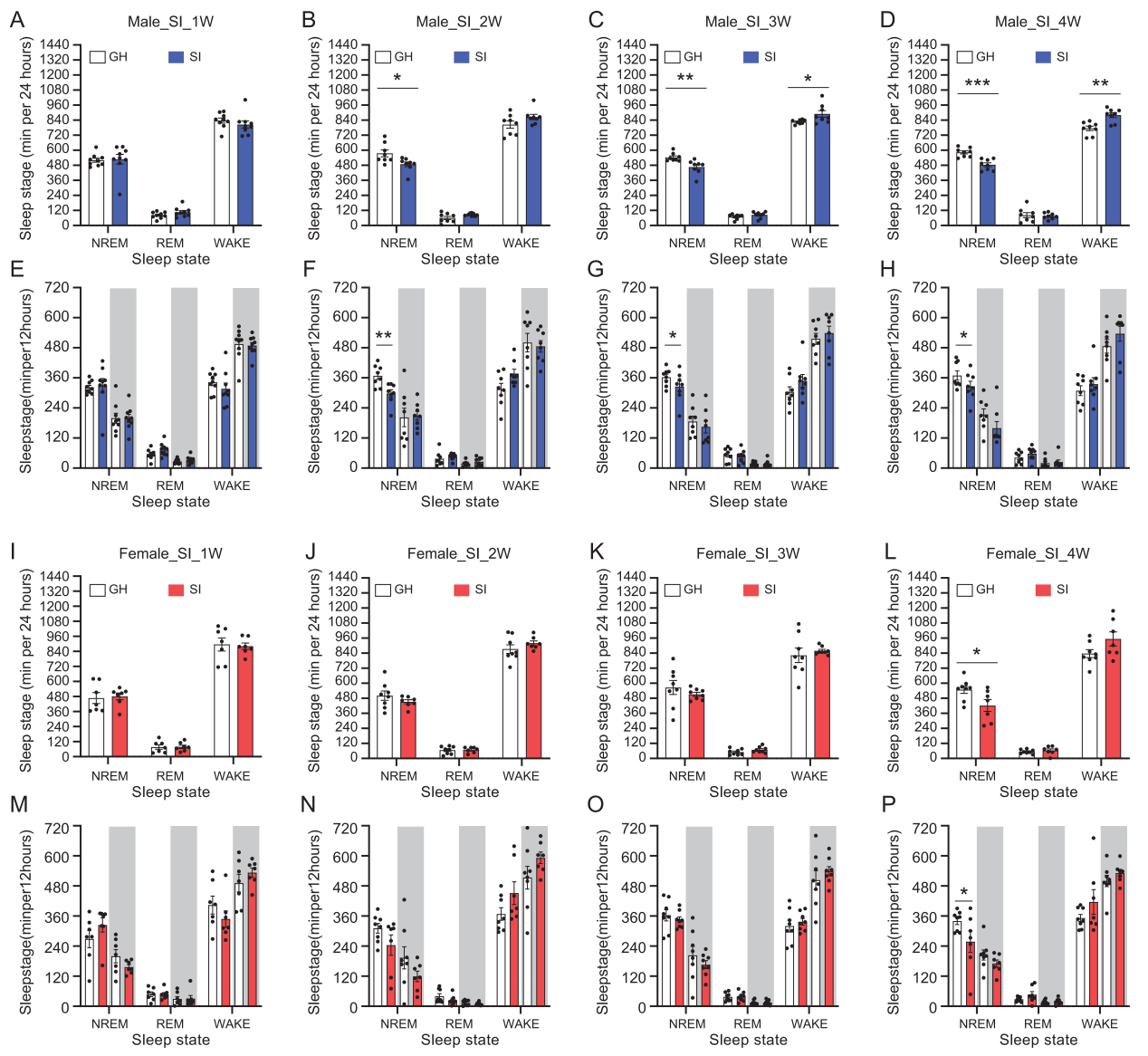


**Supplementary Fig.2.** **SI reduces the duration of NREM sleep in male mice starting from week 2, while female mice exhibit similar effects at week 4.**

(A-D) Total time (min) spent in NREM, REM, and WAKE states over 24 hours in male mice after 1 week (A), 2 weeks (B), 3 weeks (C), and 4 weeks (D) of SI.

(E-H) Time (min) spent in NREM, REM, and WAKE states over 12-hour light/dark phases in male mice after 1 week (E), 2 weeks (F), 3 weeks (G), and 4 weeks (H) of SI. White indicates the light phase and gray indicates the dark phase.

(I–J) Total time (min) spent in NREM, REM, and WAKE states over 24 hours in female mice after 1 week (I), 2 weeks (J), 3 weeks (K), and 4 weeks (L) of SI.

(M-P) Time (min) spent in NREM, REM, and WAKE states over 12-hour light/dark phases in female mice after 1 week (M), 2 weeks (N), 3 weeks (O), and 4 weeks (P) of SI.

Data are presented as mean ± SEM. Each dot represents one mouse (n = 7-9 per group). Statistical significance was assessed using an unpaired two-tailed Student’ s t-test. **p* < 0.05, ***p* < 0.01, ****p* < 0.001.


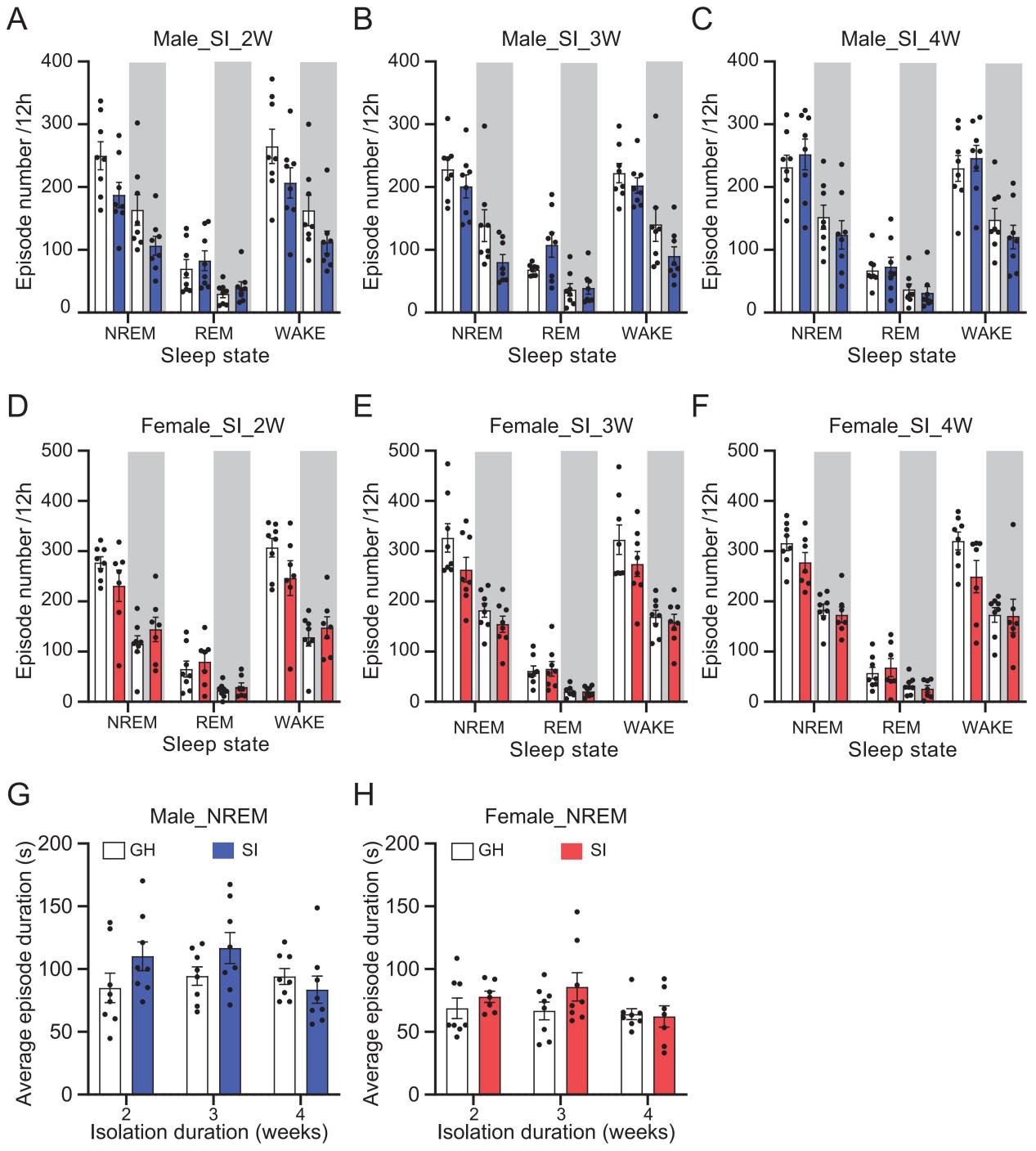


**Supplementary Fig.3. Episode number and NREM sleep fragmentation in male and female mice during light and dark phases.**

(A-C) Episode numbers were recorded in 12-hour bins (light and dark phases) for male mice after 2, 3, and 4 weeks of isolation. White indicates the light phase and gray indicates the dark phase.

(D-F) Episode numbers were recorded in 12-hour bins (light and dark phases) for female mice after 2, 3, and 4 weeks of isolation. White indicates the light phase and gray indicates the dark phase.

(G) Average NREM episode duration was expressed over a 24-hour cycle for male mice after 2, 3, and 4 weeks of isolation.

(H) Average NREM episode duration was expressed over a 24-hour cycle for female mice after 2, 3, and 4 weeks of isolation.

Date are represented as mean ± SEM.

**Supplementary Table 1. Summary of statistical analysis**

| **Figures** | **Sleep State** | **Analysis** | **Test statistic** | **P-value** | **n** |
| --- | --- | --- | --- | --- | --- |
| 1B | NREM | Two-tailed Student’s t-test | t = 2.582 | 0.0217 | 8 |
| 1C | NREM | Two-tailed Student’s t-test | t = 3.344 | 0.0048 | 8 |
| 1C | WAKE | Two-tailed Student’s t-test | t = 2.291 | 0.0380 | 8 |
| 1D | NREM | Two-tailed Student’s t-test | t = 4.171 | 0.0009 | 8 |
| 1D | WAKE | Two-tailed Student’s t-test | t = 3.360 | 0.0047 | 8 |
| 1E | NREM | Two-tailed Student’s t-test | t = 3.322 | 0.0050 | 8 |
| 1F | NREM | Two-tailed Student’s t-test | t = 2.146 | 0.0499 | 8 |
| 1G | NREM | Two-tailed Student’s t-test | t = 2.947 | 0.0106 | 8 |
| 1M | NREM | Two-tailed Student’s t-test | t = 2.327 | 0.0368 | 7-8 |
| 1P | NREM | Two-tailed Student’s t-test | t = 2.624 | 0.0210 | 7-8 |


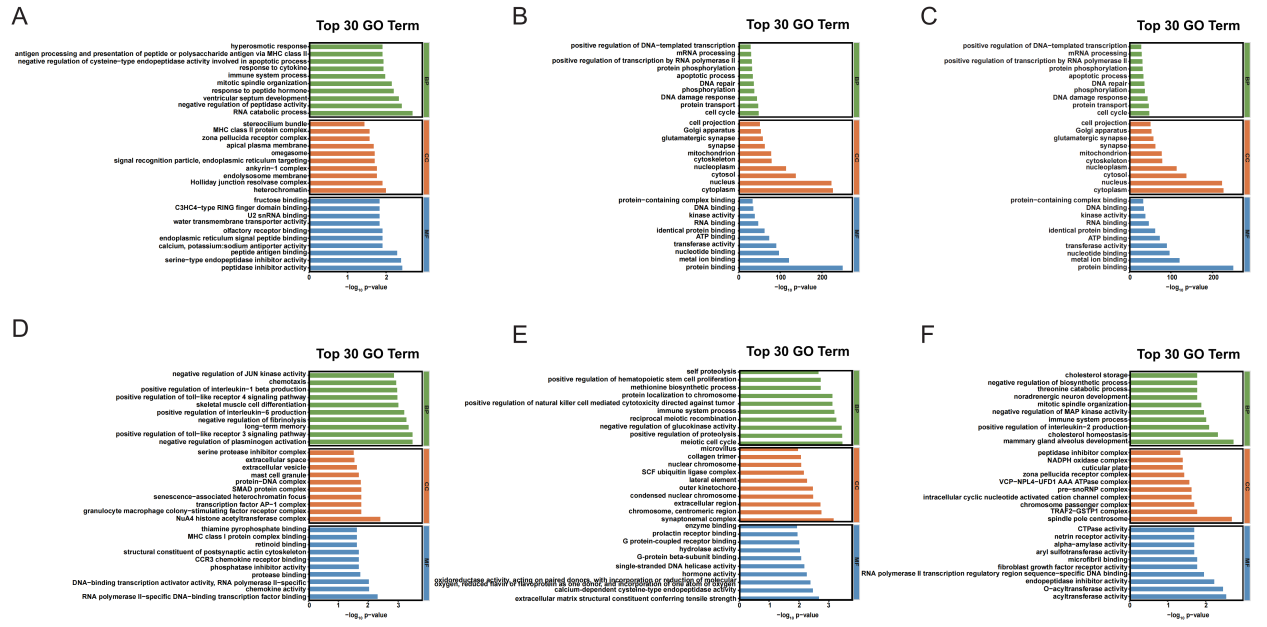


**Supplementary Fig.4. GO enrichment analysis of DEGs in male and female mice after SI**

(A-C) GO enrichment analysis of DEGs in male mice after 2, 3, and 4 weeks of isolation, highlighting the top 30 significantly enriched terms across three categories: BP, CC, and MF.

(D-F) GO enrichment analysis of DEGs in female mice after 2, 3, and 4 weeks of isolation, highlighting the top 30 significantly enriched terms across BP, CC, and MF.

BP: Biological Process; CC: Cellular Component; MF: Molecular Function.

**
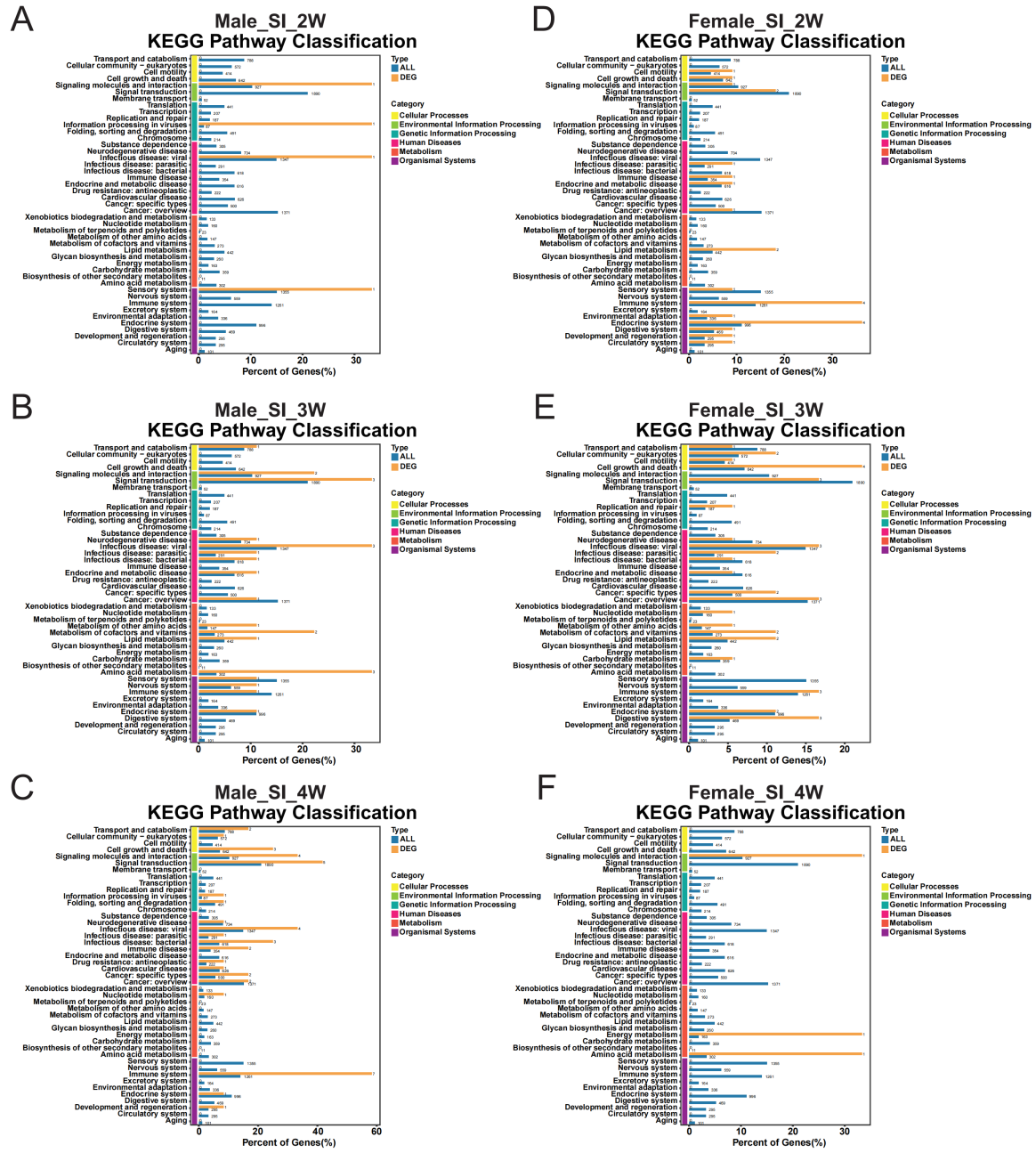
**

**Supplementary Fig.5. KEGG pathway classification of key DEGs in male and female mice after SI**

(A-C) KEGG pathway classification of key DEGs in male mice after 2, 3, and 4 weeks of isolation.

(D-F) KEGG pathway classification of key DEGs in female mice after 2, 3, and 4 weeks of isolation.


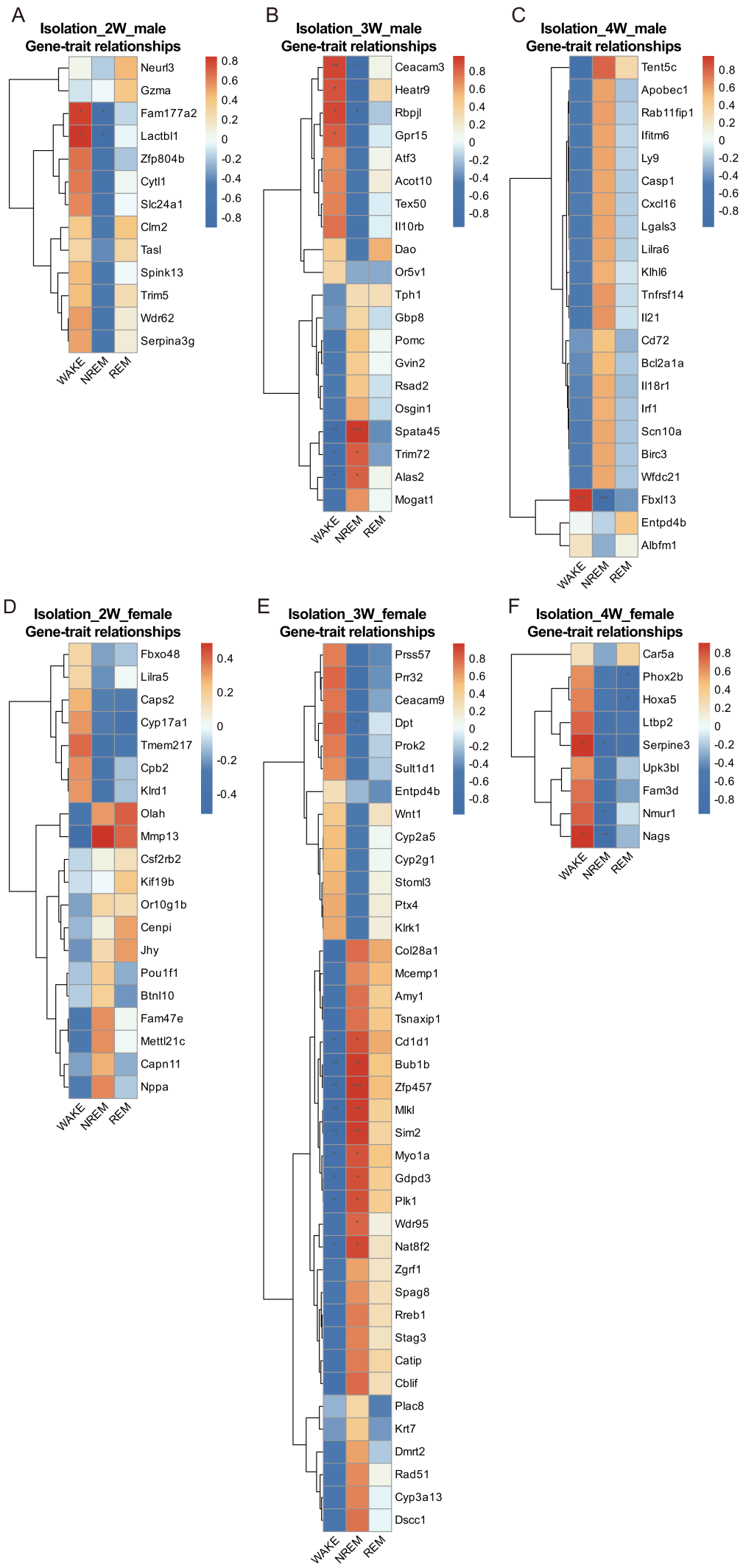


**Supplementary Fig.6. The relationship between key genes and sleep-wake traits.**

(A-C). Key genes and sleep states correlation analysis in male mice after 2, 3, and 4 weeks of isolation. Red indicates positive correlation, blue indicates negative correlation.

(D-F). Key genes and sleep states correlation analysis in female mice after 2, 3, and 4 weeks of isolation. Red indicates positive correlation, blue indicates negative correlation.

**p* < 0.05, ***p* < 0.01, and ****p* < 0.001.

**Supplementary Table 2. PCR primer sequences for RT-qPCR analysis of gene expression.**

| Genes | Forward 5′-3′ | Reverse 5′-3′ |
| --- | --- | --- |
| Lactbl1 | CAGATGCCACTACAGCACG | CCTGCCGCTGCCATAGAAG |
| Serpina3g | CTTCCCAACGGCTGGAATCTA | ACTGTCCAATCAGGCATAGCG |
| Gzma | TGCTGCCCACTGTAACGTG | GGTAGGTGAAGGATAGCCACAT |
| Spink13 | AGCTAACTGTCCAGATGTGAAAG | CCCACCGATCAATGCAAAAGA |
| Tasl | TCTTTATGTGAGCTGCAAATCCT | CCCCTGCAATATGTAAGTCGTT |
| Btnl10 | AGGTGACAGAGACTTACCTGC | ACAGGCTATTGCTGCTGAAAC |
| Mmp13 | CTTCTTCTTGTTGAGCTGGACTC | CTGTGGAGGTCACTGTAGACT |
| Cyp17a1 | GCCCAAGTCAAAGACACCTAAT | GTACCCAGGCGAAGAGAATAGA |
| Klrd1 | TCTAGGATCACTCGGTGGAGA | CACTTGTCCAGGCAAACACAG |
| Nppa | GCTTCCAGGCCATATTGGAG | GGGGGCATGACCTCATCTT |
| Il10rb | ACCTGCTTTCCCCAAAACGAA | TGAGAGAAGTCGCACTGAGTC |
| Or5v1 | ACCACCTGAATGAATTGCAGTAT | CAGCTATGGTCACCACAATGAT |
| Heatr9 | CAAATGCCAAACCTCCATGTCT | GGGATCTAACAACCCTGGTCC |
| Acot10 | GCAGCGATGAGGCTTTGGA | TGATGGATGTGTAAGGGTTCACT |
| Ceacam3 | TGCCCACCACTACCCAAGT | TGCCCACCACTACCCAAGT |
| Cyp2g1 | GGCACTTTGTTTGTCTTGCCT | TCCCAAAAACGGTATTGGTGTG |
| Sult1d1 | ATGTCTTCAGGAGGGAGTTAGTG | CATCAGGCCGGGCTTCAAA |
| Cyp2a5 | TGGTCCTGTATTCACCATCTACC | ACTACGCCATAGCCTTTGAAAA |
| Klrk1 | TGGTCCTGTATTCACCATCTACC | CAGGTTGACTGGTAGTTAGTGC |
| Cblif | CCCTCTACCTCCTAAGTGTTCTC | CTGAGTCAGTCACCGAGTTCT |
| Albfm1 | GACAGCCATGATACACCCCTG | GCTCAAGATAGTGGGTCCGTA |
| Bcl2a1a | GGCTGAGCACTACCTTCAGTA | TGGCGGTATCTATGGATTCCAC |
| Il21 | GGACCCTTGTCTGTCTGGTAG | TGTGGAGCTGATAGAAGTTCAGG |
| Wfdc21 | AAGCTAGGAGCCTTCCTTCTG | AGCACAGGTGCCTAAAAGCTG |
| Lilra6 | CCCTGGTGCTAGTAGTGACAG | GTGATAGCTCTGCGAAGACTC |
| Upk3bl | CGAATGCCCCTGTTGTTGC | AGGTAGATTGTGTGAGTCTCCC |
| Nags | CTCTAGCCAAGGCTTTGCAG | CAGCCACTCAGCGTTGGTA |
| Hoxa5 | CTCATTTTGCGGTCGCTATCC | ATCCATGCCATTGTAGCCGTA |
| Fam3d | CTGATCCGTGTTGTGGTCTTC | CAGCGTGGCAGACGAATTG |
| Phox2b | GGGCTAAGTTTCGCAAGCAG | CAGTGCTGTCGGGATCAGTG |
| 18S rRNA | AGTTCCAGCACATTTTGCGAG | TCATCCTCCGTGAGTTCTCCA |


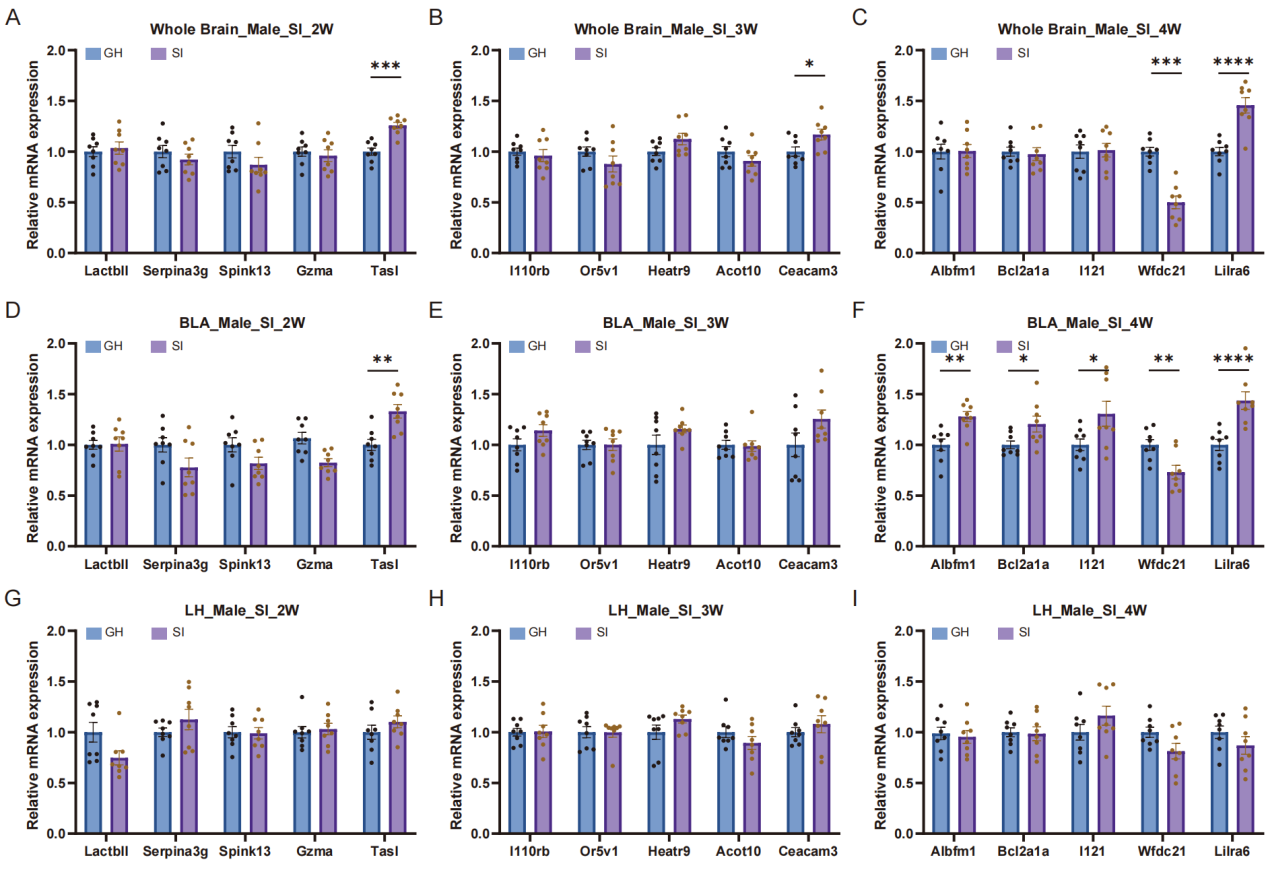


**Supplementary Fig.7.** **The relative expression levels of the top 5 key genes in the whole brain, BLA, and LH of male mice after 2, 3, and 4 weeks of isolation.**

(A-C). Relative expression of top 5 key genes in the whole brain of male mice after 2, 3, and 4 weeks of isolation.

(D-F). Relative expression of top 5 key genes in the BLA of male mice after 2, 3, and 4 weeks of isolation.

(G-I). Relative expression of top 5 key genes in the LH of male mice after 2, 3, and 4 weeks of isolation.

BLA: basolateral amygdala; LH: lateral hypothalamus

Date are represented as mean ± SEM. **p* < 0.05, ***p* < 0.01, ****p* < 0.001, and *****p* < 0.0001 .


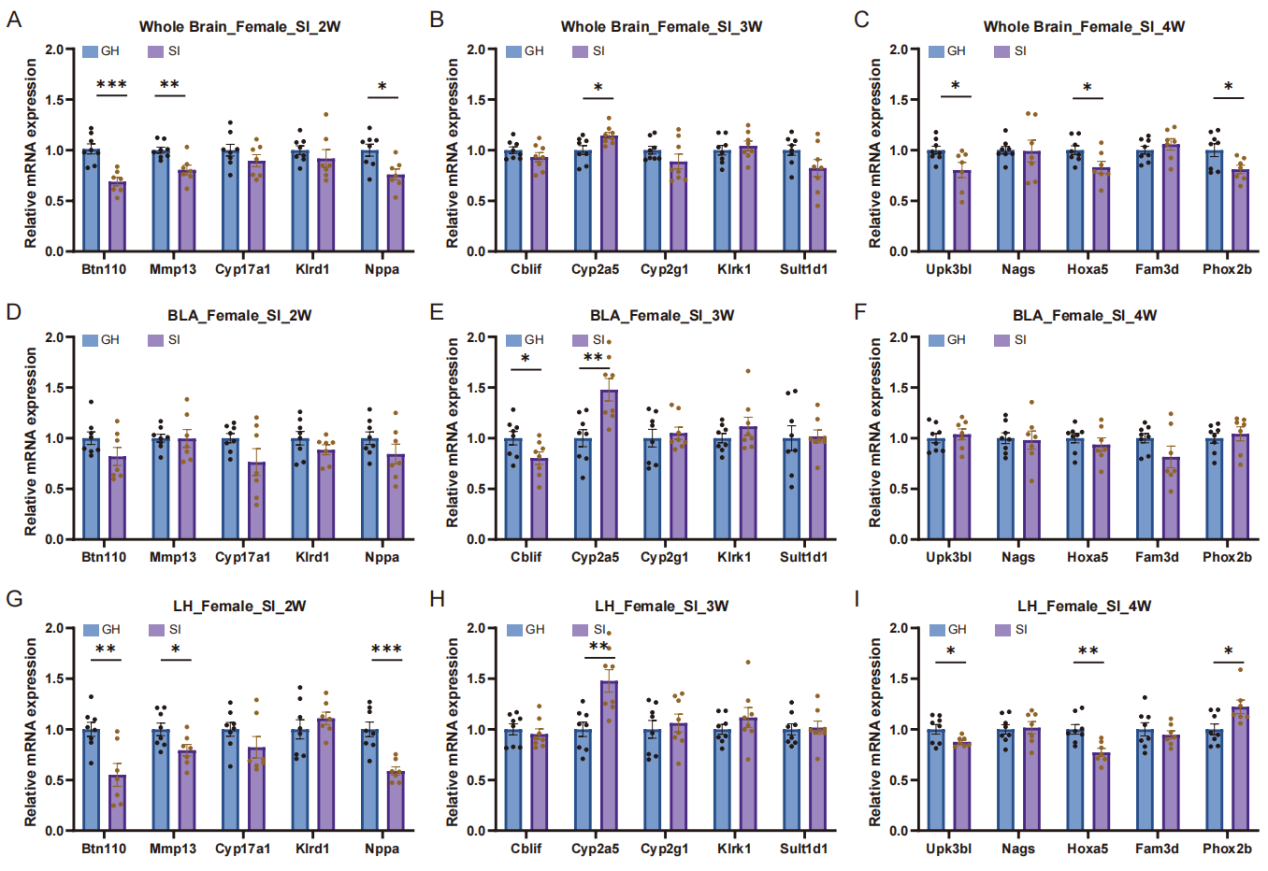


**Supplementary Fig.8.** **The relative expression levels of the top 5 key genes in the whole brain, BLA, and LH of female mice after 2, 3, and 4 weeks of isolation.**

(A-C). Relative expression of top 5 key genes in the whole brain of female mice after 2, 3, and 4 weeks of isolation.

(D-F). Relative expression of top 5 key genes in the BLA of female mice after 2, 3, and 4 weeks of isolation.

(G-I). Relative expression of top 5 key genes in the LH of female mice after 2, 3, and 4 weeks of isolation.

BLA: basolateral amygdala; LH: lateral hypothalamus

Date are represented as mean ± SEM. **p* < 0.05, ***p* < 0.01, and ****p* < 0.001.
